# The genome sequence of the spotted cranefly, *Nephrotoma appendiculata *(Pierre, 1919)

**DOI:** 10.12688/wellcomeopenres.20886.1

**Published:** 2024-02-15

**Authors:** Liam M. Crowley, Denise C. Wawman, University of Oxford and Wytham Woods Genome Acquisition Lab, Darwin Tree of Life Barcoding collective, Wellcome Sanger Institute Tree of Life Management, Samples and Laboratory team, Wellcome Sanger Institute Scientific Operations: Sequencing Operations, Wellcome Sanger Institute Tree of Life Core Informatics team, Tree of Life Core Informatics collective, Darwin Tree of Life Consortium

**Affiliations:** 1 Department of Biology, University of Oxford, Oxford, England, UK

**Keywords:** Nephrotoma appendiculata, spotted cranefly, genome sequence, chromosomal, Diptera

## Abstract

We present a genome assembly from an individual male *Nephrotoma appendiculata* (the spotted cranefly; Arthropoda; Insecta; Diptera; Tipulidae). The genome sequence is 1,138.0 megabases in span. Most of the assembly is scaffolded into 4 chromosomal pseudomolecules, including the X sex chromosome. The mitochondrial genome has also been assembled and is 17.42 kilobases in length. Gene annotation of this assembly on Ensembl identified 17,753 protein coding genes.

## Species taxonomy

Eukaryota; Metazoa; Eumetazoa; Bilateria; Protostomia; Ecdysozoa; Panarthropoda; Arthropoda; Mandibulata; Pancrustacea; Hexapoda; Insecta; Dicondylia; Pterygota; Neoptera; Endopterygota; Diptera; Nematocera; Tipulomorpha; Tipuloidea; Tipulidae; Tipulinae; *Nephrotoma*, *Nephrotoma appendiculata* (Pierre, 1919) (NCBI:txid2741127).

## Background

The Spotted or Inverted-U Tiger Cranefly *Nephrotoma appendiculata* is a member of the family Tipulidae, or long-palped craneflies, and as such, it has the fairly typical “Daddy Long Legs” shape of these Diptera. Those in the genus *Nephrotoma* have black stipes on a yellow background, earning them the name tiger craneflies, and a distinctive pattern of wing venation that distinguishes them from other genera ( Stubbs, 2021).

*Nephrotoma appendiculata* is a moderate sized cranefly with a wing length of 12–15 mm. It usually has a pale stigma, but this can be dark in a few specimens. There is a wide dull black stipe on the dorsal abdomen reaching across to the yellow sides. The determining feature is an upside-down U-shaped black mark around the base of the halteres ( Stubbs, 2021).

*Nephrotoma appendiculata* is a common grassland species with adults flying from April to early June. It is tolerant of a range of pH and moisture levels, preferring unimproved grassland with medium to long grass, on better soils, but avoiding short turf, impoverished grassland and shade ( Stubbs, 2021).

The structure of the spermatozoa of *Nephrotoma appendiculata* was found to be similar to that of several craneflies in the family Limoniidae and this has been used to support the idea that the families Tipulidae and Limoniidae should be combined ( Dallai *et al.*, 2008) but they currently remain classified into two families ( Chandler, 2023), and the final decision is likely to be based on phylogenetic analyses of DNA sequences.

The genome of the spotted cranefly, *Nephrotoma appendiculata*, was sequenced as part of the Darwin Tree of Life Project, a collaborative effort to sequence all named eukaryotic species in the Atlantic Archipelago of Britain and Ireland. Here we present a chromosomally complete genome sequence for *Nephrotoma appendiculata*, based on one male specimen from Wytham Woods, Oxfordshire, UK.

## Genome sequence report

**Figure 1.**
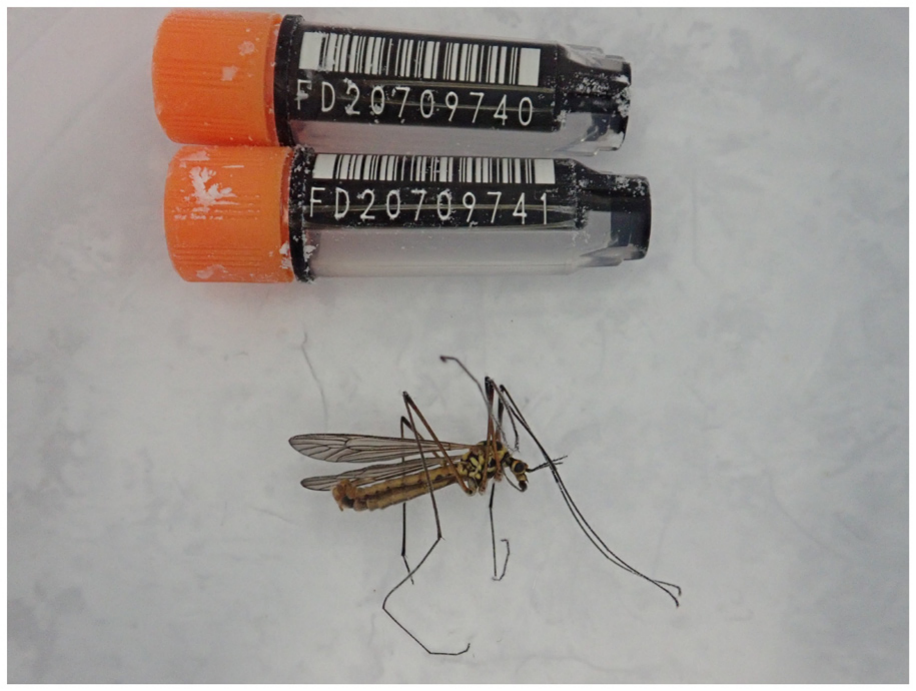
Photograph of the *Nephrotoma appendiculata* (idNepAppe1) specimen used for genome sequencing.

The genome was sequenced from one male *Nephrotoma appendiculata* ( Figure 1) collected from Wytham Woods, Oxfordshire, UK (51.76, –1.34). A total of 30-fold coverage in Pacific Biosciences single-molecule HiFi long reads was generated. Primary assembly contigs were scaffolded with chromosome conformation Hi-C data. Manual assembly curation corrected 70 missing joins or mis-joins and removed 17 haplotypic duplications, reducing the assembly length by 0.25% and the scaffold number by 12.06%, and decreasing the scaffold N50 by 45.60%.

**Figure 2.**
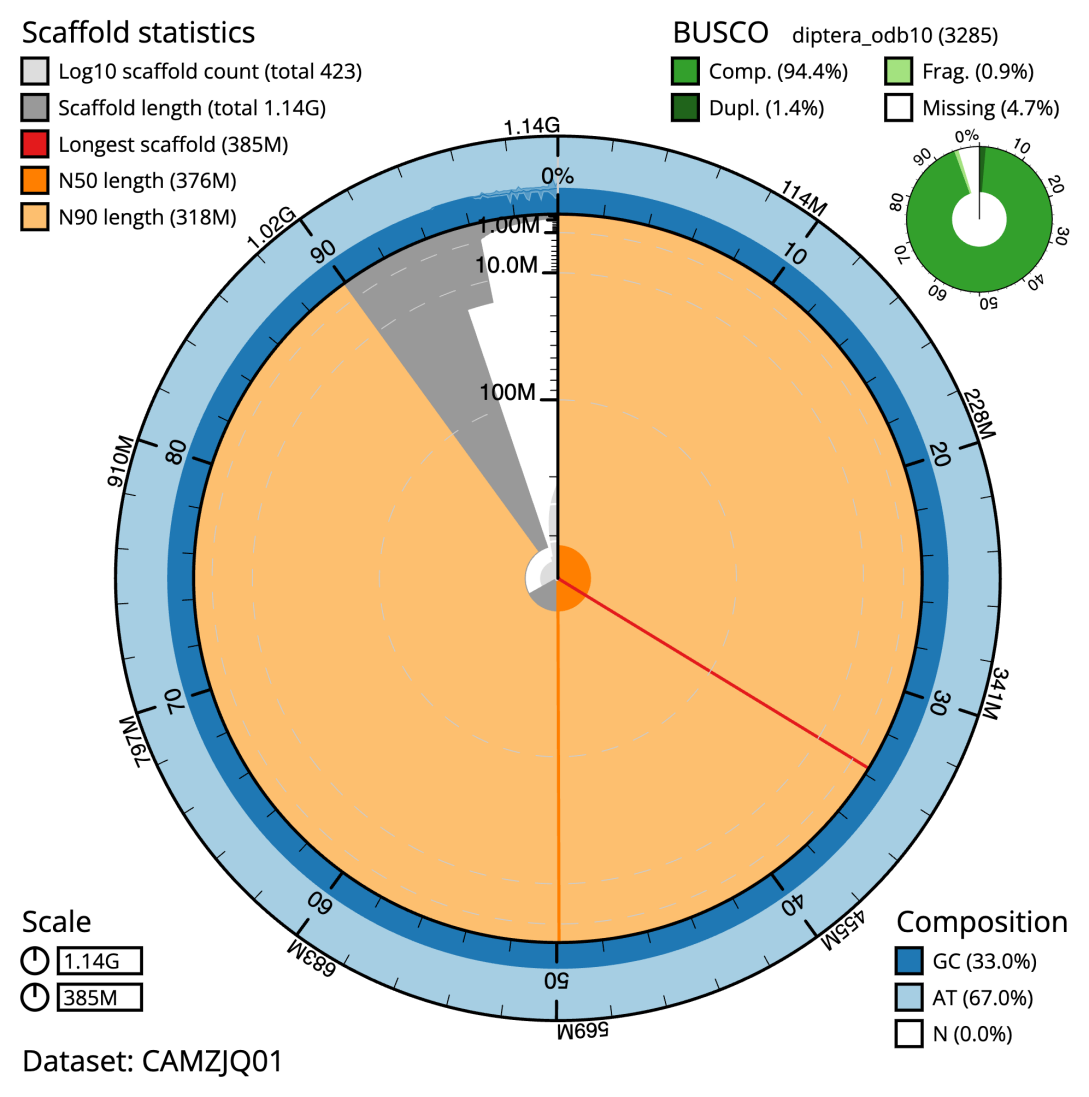
Genome assembly of *Nephrotoma appendiculata*, idNepAppe1.1: metrics. The BlobToolKit Snailplot shows N50 metrics and BUSCO gene completeness. The main plot is divided into 1,000 size-ordered bins around the circumference with each bin representing 0.1% of the 1,138,061,071 bp assembly. The distribution of scaffold lengths is shown in dark grey with the plot radius scaled to the longest scaffold present in the assembly (384,599,746 bp, shown in red). Orange and pale-orange arcs show the N50 and N90 scaffold lengths (375,927,842 and 317,755,181 bp), respectively. The pale grey spiral shows the cumulative scaffold count on a log scale with white scale lines showing successive orders of magnitude. The blue and pale-blue area around the outside of the plot shows the distribution of GC, AT and N percentages in the same bins as the inner plot. A summary of complete, fragmented, duplicated and missing BUSCO genes in the diptera_odb10 set is shown in the top right. An interactive version of this figure is available at https://blobtoolkit.genomehubs.org/view/CAMZJQ01/dataset/CAMZJQ01/snail.

**Figure 3.**
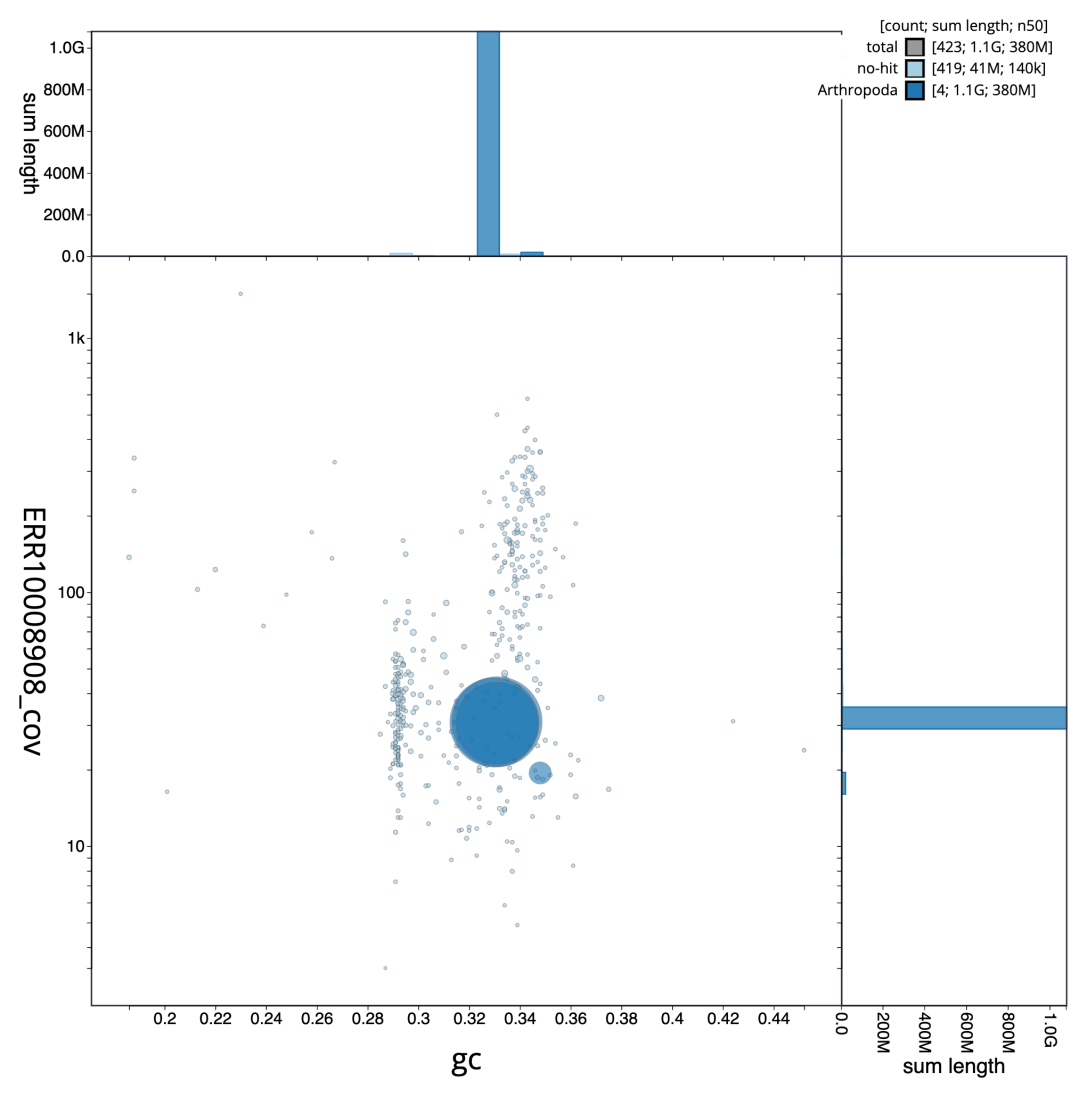
Genome assembly of *Nephrotoma appendiculata*, idNepAppe1.1: BlobToolKit GC-coverage plot. Scaffolds are coloured by phylum. Circles are sized in proportion to scaffold length. Histograms show the distribution of scaffold length sum along each axis. An interactive version of this figure is available at https://blobtoolkit.genomehubs.org/view/CAMZJQ01/dataset/CAMZJQ01/blob.

**Figure 4.**
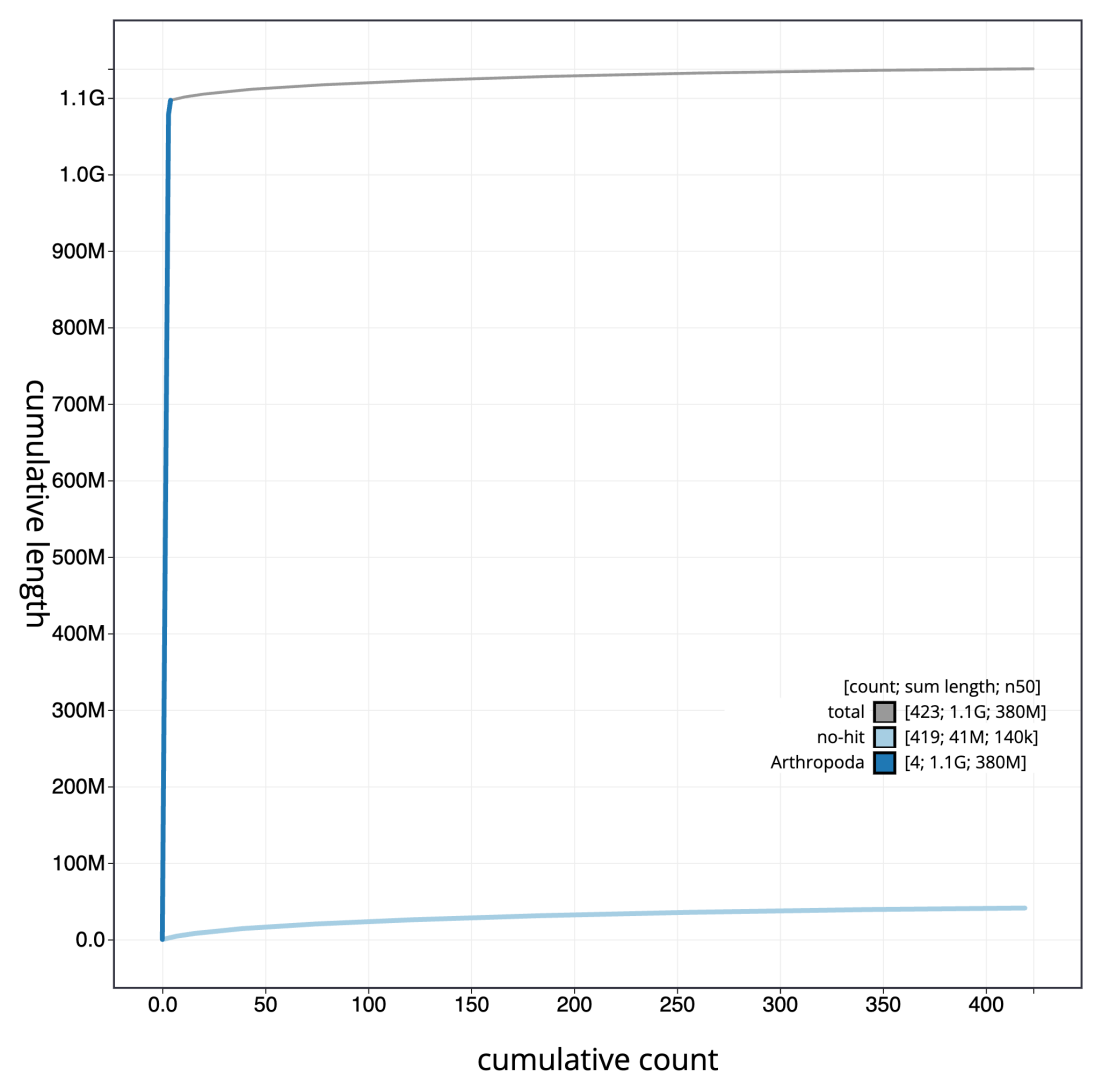
Genome assembly of *Nephrotoma appendiculata*, idNepAppe1.1: BlobToolKit cumulative sequence plot. The grey line shows cumulative length for all scaffolds. Coloured lines show cumulative lengths of scaffolds assigned to each phylum using the buscogenes taxrule. An interactive version of this figure is available at https://blobtoolkit.genomehubs.org/view/CAMZJQ01/dataset/CAMZJQ01/cumulative.

**Figure 5.**
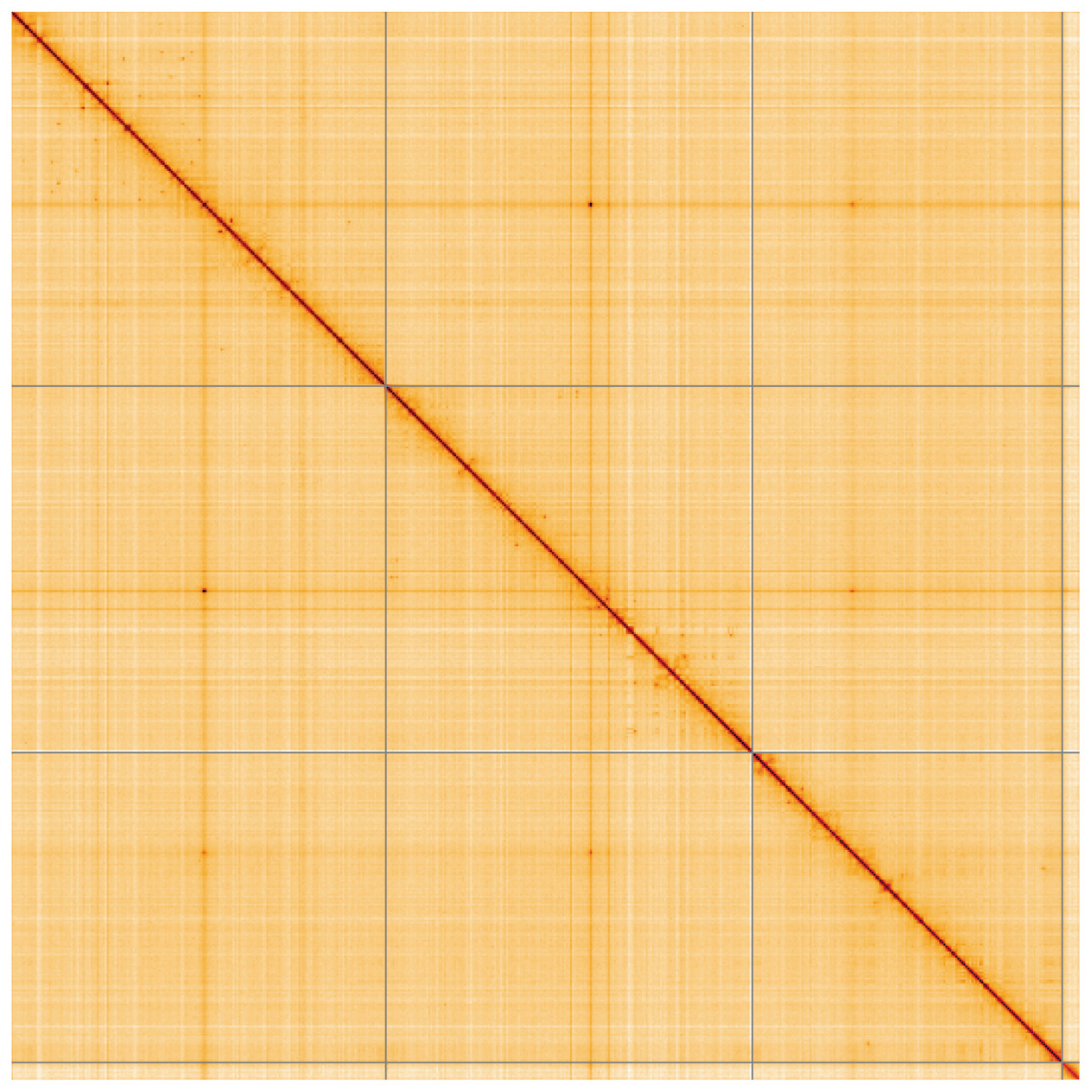
Genome assembly of *Nephrotoma appendiculata*, idNepAppe1.1: Hi-C contact map of the idNepAppe1.1 assembly, visualised using HiGlass. Chromosomes are shown in order of size from left to right and top to bottom. An interactive version of this figure may be viewed at https://genome-note-higlass.tol.sanger.ac.uk/l/?d=ENODhlutRSaWfOvEvECH7A.

The final assembly has a total length of 1138.0 Mb in 422 sequence scaffolds with a scaffold N50 of 375.9 Mb ( Table 1). The snailplot in Figure 2 provides a summary of the assembly statistics, while the distribution of assembly scaffolds on GC proportion and coverage is shown in Figure 3. The cumulative assembly plot in Figure 4 shows curves for subsets of scaffolds assigned to different phyla. Most (99.7%) of the assembly sequence was assigned to 4 chromosomal-level scaffolds, representing 3 autosomes and the X sex chromosome. Chromosome-scale scaffolds confirmed by the Hi-C data are named in order of size ( Figure 5; Table 2). While not fully phased, the assembly deposited is of one haplotype. Contigs corresponding to the second haplotype have also been deposited. The mitochondrial genome was also assembled and can be found as a contig within the multifasta file of the genome submission.

**Table 1.** Genome data for *Nephrotoma appendiculata*, idNepAppe1.1.

Project accession data		
Assembly identifier	idNepAppe1.1	
Species	*Nephrotoma appendiculata*	
Specimen	idNepAppe1	
NCBI taxonomy ID	2741127	
BioProject	PRJEB55031	
BioSample ID	SAMEA10166758	
Isolate information	idNepAppe1, male: head and thorax (DNA and Hi-C sequencing), abdomen (RNA sequencing)	
Assembly metrics *		*Benchmark*
Consensus quality (QV)	61.0	*≥ 50*
*k*-mer completeness	100.0%	*≥ 95%*
BUSCO **	C:94.4%[S:93.0%,D:1.4%], F:0.9%,M:4.7%,n:3,285	*C ≥ 95%*
Percentage of assembly mapped to chromosomes	99.7%	*≥ 95%*
Sex chromosomes	X	*localised homologous pairs*
Organelles	Mitochondrial genome: 17.42 kb	*complete single alleles*
**Raw data accessions**		
PacificBiosciences SEQUEL II	ERR10008908	
Hi-C Illumina	ERR10015065	
PolyA RNA-Seq Illumina	ERR10378025	
**Genome assembly**		
Assembly accession	GCA_947310385.1	
*Accession of alternate haplotype*	GCA_947311015.1	
Span (Mb)	1,138.0	
Number of contigs	1102	
Contig N50 length (Mb)	3.2	
Number of scaffolds	422	
Scaffold N50 length (Mb)	375.9	
Longest scaffold (Mb)	384.6	
**Genome annotation**		
Number of protein-coding genes	14,126	
Number of non-coding genes	3,241	
Number of gene transcripts	24,340	

* Assembly metric benchmarks are adapted from column VGP-2020 of “Table 1: Proposed standards and metrics for defining genome assembly quality” from ( Rhie *et al.*, 2021). ** BUSCO scores based on the diptera_odb10 BUSCO set using version 5.3.2. C = complete [S = single copy, D = duplicated], F = fragmented, M = missing, n = number of orthologues in comparison. A full set of BUSCO scores is available at https://blobtoolkit.genomehubs.org/view/CAMZJQ01/dataset/CAMZJQ01/busco.

**Table 2.** Chromosomal pseudomolecules in the genome assembly of Nephrotoma appendiculata, idNepAppe1.

INSDC accession	Chromosome	Length (Mb)	GC%
OX371223.1	1	384.6	33.0
OX371224.1	2	375.93	33.0
OX371225.1	3	317.76	33.0
OX371226.1	X	18.65	35.0
OX371227.1	MT	0.02	23.0

The estimated Quality Value (QV) of the final assembly is 61.0 with *k*-mer completeness of 100.0%, and the assembly has a BUSCO v5.3.2 completeness of 94.4% (single = 93.0%, duplicated = 1.4%), using the diptera_odb10 reference set ( *n* = 3,285).

Metadata for specimens, barcode results, spectra estimates, sequencing runs, contaminants and pre-curation assembly statistics are given at https://links.tol.sanger.ac.uk/species/2741127.

## Genome annotation report

The *Nephrotoma appendiculata* genome assembly (GCA_947310385.1) was annotated using the Ensembl rapid annotation pipeline ( Table 1; https://rapid.ensembl.org/Nephrotoma_appendiculata_GCA_947310385.1/Info/Index). The resulting annotation includes 24,340 transcribed mRNAs from 14,126 protein-coding genes and 3,241 non-coding genes.

## Methods

### Sample acquisition and nucleic acid extraction

A male *Nephrotoma appendiculata* (specimen ID Ox001277, ToLID idNepAppe1) was netted in Wytham Woods, Oxfordshire (biological vice-county Berkshire), UK (latitude 51.76, longitude –1.34) on 2021-04-23. The specimen was collected and identified by Liam Crowley (University of Oxford) and preserved on dry ice.

Protocols developed by the Wellcome Sanger Institute (WSI) Tree of Life core laboratory have been deposited on protocols.io ( Denton *et al.*, 2023b). The workflow for high molecular weight (HMW) DNA extraction at the WSI includes a sequence of core procedures: sample preparation; sample homogenisation, DNA extraction, fragmentation, and clean-up. In sample preparation, the idNepAppe1 sample was weighed and dissected on dry ice ( Jay *et al.*, 2023). Tissue from the head and thorax was homogenised using a PowerMasher II tissue disruptor ( Denton *et al.*, 2023a). HMW DNA was extracted in the WSI Scientific Operations core using the Automated MagAttract v2 protocol ( Oatley *et al.*, 2023). HMW DNA was sheared into an average fragment size of 12–20 kb in a Megaruptor 3 system with speed setting 31 ( Bates *et al.*, 2023). Sheared DNA was purified by solid-phase reversible immobilisation ( Strickland *et al.*, 2023): in brief, the method employs a 1.8X ratio of AMPure PB beads to sample to eliminate shorter fragments and concentrate the DNA. The concentration of the sheared and purified DNA was assessed using a Nanodrop spectrophotometer and Qubit Fluorometer and Qubit dsDNA High Sensitivity Assay kit. Fragment size distribution was evaluated by running the sample on the FemtoPulse system.

RNA was extracted from abdomen tissue of idNepAppe1 in the Tree of Life Laboratory at the WSI using the RNA Extraction: Automated MagMax™ *mir*Vana protocol ( do Amaral *et al.*, 2023). The RNA concentration was assessed using a Nanodrop spectrophotometer and a Qubit Fluorometer using the Qubit RNA Broad-Range Assay kit. Analysis of the integrity of the RNA was done using the Agilent RNA 6000 Pico Kit and Eukaryotic Total RNA assay.

### Sequencing

Pacific Biosciences HiFi circular consensus DNA sequencing libraries were constructed according to the manufacturers’ instructions. Poly(A) RNA-Seq libraries were constructed using the NEB Ultra II RNA Library Prep kit. DNA and RNA sequencing was performed by the Scientific Operations core at the WSI on Pacific Biosciences SEQUEL II (HiFi) and Illumina NovaSeq 6000 (RNA-Seq) instruments. Hi-C data were also generated from remaining head and thorax tissue of idNepAppe1 using the Arima2 kit and sequenced on the Illumina NovaSeq 6000 instrument.

### Genome assembly, curation and evaluation

Assembly was carried out with Hifiasm ( Cheng *et al.*, 2021) and haplotypic duplication was identified and removed with purge_dups ( Guan *et al.*, 2020). The assembly was then scaffolded with Hi-C data ( Rao *et al.*, 2014) using YaHS ( Zhou *et al.*, 2023). The assembly was checked for contamination and corrected as described previously ( Howe *et al.*, 2021). Manual curation was performed using HiGlass ( Kerpedjiev *et al.*, 2018) and Pretext ( Harry, 2022). The mitochondrial genome was assembled using MitoHiFi ( Uliano-Silva *et al.*, 2023), which runs MitoFinder ( Allio *et al.*, 2020) or MITOS ( Bernt *et al.*, 2013) and uses these annotations to select the final mitochondrial contig and to ensure the general quality of the sequence.

A Hi-C map for the final assembly was produced using bwa-mem2 ( Vasimuddin *et al.*, 2019) in the Cooler file format ( Abdennur & Mirny, 2020). To assess the assembly metrics, the *k*-mer completeness and QV consensus quality values were calculated in Merqury ( Rhie *et al.*, 2020). This work was done using Nextflow ( Di Tommaso *et al.*, 2017) DSL2 pipelines “sanger-tol/readmapping” ( Surana *et al.*, 2023a) and “sanger-tol/genomenote” ( Surana *et al.*, 2023b). The genome was analysed within the BlobToolKit environment ( Challis *et al.*, 2020) and BUSCO scores ( Manni *et al.*, 2021; Simão *et al.*, 2015) were calculated.

Table 3 contains a list of relevant software tool versions and sources.

**Table 3.** Software tools: versions and sources.

Software tool	Version	Source
BlobToolKit	4.1.7	https://github.com/blobtoolkit/blobtoolkit
BUSCO	5.3.2	https://gitlab.com/ezlab/busco
Hifiasm	0.16.1-r375	https://github.com/chhylp123/hifiasm
HiGlass	1.11.6	https://github.com/higlass/higlass
Merqury	MerquryFK	https://github.com/thegenemyers/MERQURY.FK
MitoHiFi	2	https://github.com/marcelauliano/MitoHiFi
PretextView	0.2	https://github.com/wtsi-hpag/PretextView
purge_dups	1.2.3	https://github.com/dfguan/purge_dups
sanger-tol/genomenote	v1.0	https://github.com/sanger-tol/genomenote
sanger-tol/readmapping	1.1.0	https://github.com/sanger-tol/readmapping/tree/1.1.0
YaHS	yahs-1.1.91eebc2	https://github.com/c-zhou/yahs

### Genome annotation

The Ensembl gene annotation system ( Aken *et al.*, 2016) was used to generate annotation for the *Nephrotoma appendiculata* assembly (GCA_947310385.1). Annotation was created primarily through alignment of transcriptomic data to the genome, with gap filling via protein-to-genome alignments of a select set of proteins from UniProt ( UniProt Consortium, 2019).

### Wellcome Sanger Institute – Legal and Governance

The materials that have contributed to this genome note have been supplied by a Darwin Tree of Life Partner. The submission of materials by a Darwin Tree of Life Partner is subject to the **‘Darwin Tree of Life Project Sampling Code of Practice’**, which can be found in full on the Darwin Tree of Life website here. By agreeing with and signing up to the Sampling Code of Practice, the Darwin Tree of Life Partner agrees they will meet the legal and ethical requirements and standards set out within this document in respect of all samples acquired for, and supplied to, the Darwin Tree of Life Project.

Further, the Wellcome Sanger Institute employs a process whereby due diligence is carried out proportionate to the nature of the materials themselves, and the circumstances under which they have been/are to be collected and provided for use. The purpose of this is to address and mitigate any potential legal and/or ethical implications of receipt and use of the materials as part of the research project, and to ensure that in doing so we align with best practice wherever possible. The overarching areas of consideration are:

   •  Ethical review of provenance and sourcing of the material

   •  Legality of collection, transfer and use (national and international)

Each transfer of samples is further undertaken according to a Research Collaboration Agreement or Material Transfer Agreement entered into by the Darwin Tree of Life Partner, Genome Research Limited (operating as the Wellcome Sanger Institute), and in some circumstances other Darwin Tree of Life collaborators.

## Data Availability

European Nucleotide Archive: *Nephrotoma appendiculata* (spotted cranefly). Accession number PRJEB55031; https://identifiers.org/ena.embl/PRJEB55031 ( Wellcome Sanger Institute, 2022). The genome sequence is released openly for reuse. The *Nephrotoma appendiculata* genome sequencing initiative is part of the Darwin Tree of Life (DToL) project. All raw sequence data and the assembly have been deposited in INSDC databases. Raw data and assembly accession identifiers are reported in Table 1.

## Author information

Members of the University of Oxford and Wytham Woods Genome Acquisition Lab are listed here: https://doi.org/10.5281/zenodo.7125292.

Members of the Darwin Tree of Life Barcoding collective are listed here: https://doi.org/10.5281/zenodo.4893703.

Members of the Wellcome Sanger Institute Tree of Life Management, Samples and Laboratory team are listed here: https://doi.org/10.5281/zenodo.10066175.

Members of Wellcome Sanger Institute Scientific Operations: Sequencing Operations are listed here: https://doi.org/10.5281/zenodo.10043364.

Members of the Wellcome Sanger Institute Tree of Life Core Informatics team are listed here: https://doi.org/10.5281/zenodo.10066637.

Members of the Tree of Life Core Informatics collective are listed here: https://doi.org/10.5281/zenodo.5013541.

Members of the Darwin Tree of Life Consortium are listed here: https://doi.org/10.5281/zenodo.4783558.

## References

[ref-1] Abdennur N Mirny LA : Cooler: Scalable storage for Hi-C data and other genomically labeled arrays. *Bioinformatics.* 2020;36(1):311–316. 10.1093/bioinformatics/btz540 31290943 PMC8205516

[ref-2] Aken BL Ayling S Barrell D : The Ensembl gene annotation system. *Database (Oxford).* 2016;2016: baw093. 10.1093/database/baw093 27337980 PMC4919035

[ref-3] Allio R Schomaker-Bastos A Romiguier J : MitoFinder: Efficient automated large‐scale extraction of mitogenomic data in target enrichment phylogenomics. *Mol Ecol Resour.* 2020;20(4):892–905. 10.1111/1755-0998.13160 32243090 PMC7497042

[ref-4] Bates A Clayton-Lucey I Howard C : Sanger Tree of Life HMW DNA Fragmentation: Diagenode Megaruptor®3 for LI PacBio. *protocols.io.* 2023. 10.17504/protocols.io.81wgbxzq3lpk/v1

[ref-5] Bernt M Donath A Jühling F : MITOS: Improved *de novo* metazoan mitochondrial genome annotation. *Mol Phylogenet Evol.* 2013;69(2):313–319. 10.1016/j.ympev.2012.08.023 22982435

[ref-6] Challis R Richards E Rajan J : BlobToolKit - interactive quality assessment of genome assemblies. *G3 (Bethesda).* 2020;10(4):1361–1374. 10.1534/g3.119.400908 32071071 PMC7144090

[ref-7] Chandler P : Checklist of Diptera of the British Isles.2023. Reference Source

[ref-8] Cheng H Concepcion GT Feng X : Haplotype-resolved *de novo* assembly using phased assembly graphs with hifiasm. *Nat Methods.* 2021;18(2):170–175. 10.1038/s41592-020-01056-5 33526886 PMC7961889

[ref-9] Dallai R Lombardo BM Mercati D : Sperm structure of Limoniidae and their phylogenetic relationship with Tipulidae ( *Diptera, Nematocera*). *Arthropod Struct Dev.* 2008;37(1):81–92. 10.1016/j.asd.2007.05.002 18089129

[ref-10] Denton A Oatley G Cornwell C : Sanger Tree of Life Sample Homogenisation: PowerMash. *protocols.io.* 2023a. 10.17504/protocols.io.5qpvo3r19v4o/v1

[ref-11] Denton A Yatsenko H Jay J : Sanger Tree of Life Wet Laboratory Protocol Collection. *protocols.io.* 2023b. 10.17504/protocols.io.8epv5xxy6g1b/v1

[ref-12] Di Tommaso P Chatzou M Floden EW : Nextflow enables reproducible computational workflows. *Nat Biotechnol.* 2017;35(4):316–319. 10.1038/nbt.3820 28398311

[ref-13] do Amaral RJV Bates A Denton A : Sanger Tree of Life RNA Extraction: Automated MagMax ^TM^ mirVana. *protocols.io.* 2023. 10.17504/protocols.io.6qpvr36n3vmk/v1

[ref-14] Guan D McCarthy SA Wood J : Identifying and removing haplotypic duplication in primary genome assemblies. *Bioinformatics.* 2020;36(9):2896–2898. 10.1093/bioinformatics/btaa025 31971576 PMC7203741

[ref-15] Harry E : PretextView (Paired REad TEXTure Viewer): A desktop application for viewing pretext contact maps.2022; [Accessed 19 October 2022]. Reference Source

[ref-16] Howe K Chow W Collins J : Significantly improving the quality of genome assemblies through curation. *GigaScience.* Oxford University Press,2021;10(1): giaa153. 10.1093/gigascience/giaa153 33420778 PMC7794651

[ref-17] Jay J Yatsenko H Narváez-Gómez JP : Sanger Tree of Life Sample Preparation: Triage and Dissection. *protocols.io.* 2023. 10.17504/protocols.io.x54v9prmqg3e/v1

[ref-18] Kerpedjiev P Abdennur N Lekschas F : HiGlass: web-based visual exploration and analysis of genome interaction maps. *Genome Biol.* 2018;19(1): 125. 10.1186/s13059-018-1486-1 30143029 PMC6109259

[ref-19] Manni M Berkeley MR Seppey M : BUSCO update: Novel and streamlined workflows along with broader and deeper phylogenetic coverage for scoring of eukaryotic, prokaryotic, and viral genomes. *Mol Biol Evol.* 2021;38(10):4647–4654. 10.1093/molbev/msab199 34320186 PMC8476166

[ref-20] Oatley G Denton A Howard C : Sanger Tree of Life HMW DNA Extraction: Automated MagAttract v.2. *protocols.io.* 2023. 10.17504/protocols.io.kxygx3y4dg8j/v1

[ref-21] Rao SSP Huntley MH Durand NC : A 3D map of the human genome at kilobase resolution reveals principles of chromatin looping. *Cell.* 2014;159(7):1665–1680. 10.1016/j.cell.2014.11.021 25497547 PMC5635824

[ref-22] Rhie A McCarthy SA Fedrigo O : Towards complete and error-free genome assemblies of all vertebrate species. *Nature.* 2021;592(7856):737–746. 10.1038/s41586-021-03451-0 33911273 PMC8081667

[ref-23] Rhie A Walenz BP Koren S : Merqury: Reference-free quality, completeness, and phasing assessment for genome assemblies. *Genome Biol.* 2020;21(1): 245. 10.1186/s13059-020-02134-9 32928274 PMC7488777

[ref-24] Simão FA Waterhouse RM Ioannidis P : BUSCO: assessing genome assembly and annotation completeness with single-copy orthologs. *Bioinformatics.* 2015;31(19):3210–3212. 10.1093/bioinformatics/btv351 26059717

[ref-25] Strickland M Cornwell C Howard C : Sanger Tree of Life Fragmented DNA clean up: Manual SPRI. *protocols.io.* 2023. 10.17504/protocols.io.kxygx3y1dg8j/v1

[ref-26] Stubbs AE : British craneflies.British Entomological and Natural History Society,2021. Reference Source

[ref-27] Surana P Muffato M Qi G : sanger-tol/readmapping: sanger-tol/readmapping v1.1.0 - Hebridean Black (1.1.0). *Zenodo.* 2023a. Reference Source

[ref-28] Surana P Muffato M Sadasivan Baby C : sanger-tol/genomenote (v1.0.dev). *Zenodo.* 2023b. 10.5281/zenodo.6785935

[ref-29] Uliano-Silva M Ferreira JGRN Krasheninnikova K : MitoHiFi: a python pipeline for mitochondrial genome assembly from PacBio high fidelity reads. *BMC Bioinformatics.* 2023;24(1): 288. 10.1186/s12859-023-05385-y 37464285 PMC10354987

[ref-30] UniProt Consortium: UniProt: a worldwide hub of protein knowledge. *Nucleic Acids Res.* 2019;47(D1):D506–D515. 10.1093/nar/gky1049 30395287 PMC6323992

[ref-31] Vasimuddin Md Misra S Li H : Efficient Architecture-Aware Acceleration of BWA-MEM for Multicore Systems. In: *2019 IEEE International Parallel and Distributed Processing Symposium (IPDPS)*. IEEE,2019;314–324. 10.1109/IPDPS.2019.00041

[ref-33] Wellcome Sanger Institute: The genome sequence of the spotted cranefly, Nephrotoma appendiculata (Pierre, 1919). European Nucleotide Archive, [dataset], accession number PRJEB55031.2022.

[ref-32] Zhou C McCarthy SA Durbin R : YaHS: yet another Hi-C scaffolding tool. *Bioinformatics.* 2023;39(1): btac808. 10.1093/bioinformatics/btac808 36525368 PMC9848053

