# Supplementary material for: The genome sequence of the spotted cranefly, Nephrotoma appendiculata (Pierre, 1919)
Source: Wellcome Open Res. 2024 Feb 15;9:38. [Version 1] doi: 10.12688/wellcomeopenres.20886.1 (PMC11109560; doi:10.12688/wellcomeopenres.20886.1)
Supplement: Peer review history

# Reviewer report: Reviewer response for version 1

**DOI:** 10.21956/wellcomeopenres.23110.r81550

**Published:** 2024-05-21

**Authors:** Hans-Peter Fuehrer

**Affiliations:** 1 University of Veterinary Medicine Vienna, Vienna, Austria

The authors present a genome assembly from an individual spotted cranefly ( *Nephrotoma appendiculata*; Tipulidae). Methods are described well. Overall the manuscript is of relevance and good quality.

 Only two minor corrections are recommended:

- Abstract and Tab.1: Change 1,138.0 to 1,138
- Change to 1,102 at number of contigs in Tab 1.

Are sufficient details of methods and materials provided to allow replication by others?

Yes

Is the rationale for creating the dataset(s) clearly described?

Yes

Are the datasets clearly presented in a useable and accessible format?

Yes

Are the protocols appropriate and is the work technically sound?

Yes

Reviewer Expertise:

Parasitology, Entomology

I confirm that I have read this submission and believe that I have an appropriate level of expertise to confirm that it is of an acceptable scientific standard.

# Reviewer report: Reviewer response for version 1

**DOI:** 10.21956/wellcomeopenres.23110.r78182

**Published:** 2024-04-12

**Authors:** Adam James Reid

**Affiliations:** 1 University of Cambridge, Cambridge, UK

This genome note represents a very high quality genome assembly of the spotted cranefly, *Nephrotoma appendiculata*. The standards of methodological reporting and the availability of data and code are excellent. The authors have taken pains to acknowledge the large number of people involved in the (Darwin) Tree of Life endeavour. There are minor issues with some of the presentation, which could be improved in places.

 In the section *Genome sequence report,* I assume that “(51.76, –1.34)” is a geographical reference, but the frame of reference is not given. Perhaps it could say “latitude = 51.76, longitude = –1.34”.

 Also in the section *Genome sequence report*. I wanted to check that this phrase is correct: “and decreasing the scaffold N50 by 45.60%”, i.e. that after manual assembly the scaffold N50 reduced, presumably due to the breaking of very large scaffolds. The data wasn’t there for me to confirm.

 I’m afraid that even though I have seen them multiple times before I do not find the snail plots at all straightforward to understand. While I understand the desire to capture diverse and complicated information in a plot that can be automatically generated, I don’t think the majority of readers will get much from this. Why are there three different blue colours for the GC content on the smaller contigs? Why is there not more red representing the largest contig and dark orange representing the N50? The assembly for the Tiger Cranefly  (Sivell O, et.al., 2023 [Ref 1]) is quite similar, but the plots look very different. Is it possible to interpret the pale grey spiral and which orders of magnitude are represented by the white lines? The immediate solution to these problems would be to add more information to the legend to better guide the reader through what they are seeing.

 In Table 1, the benchmark column is generally very useful for giving the reader a good idea of what is expected of a good assembly, however the values in the *Sex Chromosomes* and *Organelles* rows don’t seem to make sense. We at least need a better explanation of what the possible values might be. Are the benchmarks met in these cases?

Are sufficient details of methods and materials provided to allow replication by others?

Yes

Is the rationale for creating the dataset(s) clearly described?

Yes

Are the datasets clearly presented in a useable and accessible format?

Yes

Are the protocols appropriate and is the work technically sound?

Yes

Reviewer Expertise:

Bioinformatics, genomics, transcriptomics, epigenomics, parasitology, developmental biology

I confirm that I have read this submission and believe that I have an appropriate level of expertise to confirm that it is of an acceptable scientific standard.

## References

[1] : The genome sequence of a Tiger Cranefly, Nephrotoma flavescens (Linnaeus, 1758). *Wellcome Open Res* .2023;8: 10.12688/wellcomeopenres.19203.1 148 10.12688/wellcomeopenres.19203.1 37275324 PMC10238818
